# Hypertensive response to exercise, hypertension and heart failure with preserved ejection fraction (HFpEF)—a continuum of disease?

**DOI:** 10.1007/s00508-023-02195-3

**Published:** 2023-04-17

**Authors:** Patrick Wiech, Laura Würzburger, Valentina A. Rossi, Stefano Caselli, Christian M. Schmied, David Niederseer

**Affiliations:** 1 Department of Cardiology, University Hospital Zurich, University Heart Center Zurich, University of Zurich, Rämistrasse 100, 8091 Zurich, Switzerland; 2 Hirslanden, Klinik im Park, Cardiovascular Center Zurich, Zurich, Switzerland

**Keywords:** HRE, HFpEF, Arterial Hypertension, Heart Failure

## Abstract

**Introduction:**

Heart failure with preserved ejection fraction (HFpEF) has been shown to be a long-term consequence of uncontrolled arterial hypertension (aHT). Other than that, hypertensive response to exercise (HRE) precedes aHT. We aim to evaluate the available evidence for a continuum of HRE, aHT and HFpEF.

**Methods:**

A literature search on PubMed was conducted to assembly the most recent data on the topic. After collecting the data, a qualitative analysis was instrumented.

**Results:**

10 studies including 16,165 subjects were analyzed with respect to the association between HRE and the future risk of developing aHT. With the exception of one study, all reported on a positive association between HRE and the future development of aHT despite methodological issues related to different definitions for HRE. Furthermore, HRE was associated with an increased risk of coronary artery disease. Moreover, we analysed 6 studies including overall 1366 subjects investigating the association between HRE and HFpEF. In these studies, increased left atrial volume index (LAVI), elevated E/e’ (as surrogate parameters of increased LV end-diastolic filling pressure and of diastolic dysfunction) and higher LV mass index have been proposed as independent predictor of HRE in patients with no known HFpEF diagnosis.

**Discussion and conclusion:**

The literature search revealed suggestive data on a connection of HRE, aHT and HFpEF. HRE seems to be an independent risk factor for aHT and aHT in turn is one of the main risk factors for HFpEF. However, further research is needed to improve our knowledge of a possible continuum of disease.

## Introduction

The global prevalence of heart failure (HF) ranges between 1–2% in developed countries and, despite outcome improvement over the past 30 years, HF still entails overall a poor prognosis, comparable to cancer [1–3]. The latest European data on outcomes in patients with HF report on an all-cause mortality over 12 months of 14% in hospitalized and 7% in out-patient patients [4]. As such, there is great need for further improvement in HF prevention, diagnosis, and treatment. The newly published guidelines on HF of the European Society of Cardiology classify HF in three distinct subcategories of HF: HF with reduced ejection fraction (HFrEF), defined as a LVEF (left ventricular ejection fraction) of $\leq$40%, HF with mildly-reduced EF (HFmrEF), defined as LVEF 41–49%, and HF with preserved EF (HFpEF), defined as LVEF $\geq$50% [1]. Currently, HF is defined as a clinical syndrome consisting of symptoms that may be accompanied by signs and which is due to structural or functional heart anomalies resulting in elevated intracardiac filling pressures and/or inadequate cardiac output at rest and/or during exercise [5]. The diagnosis of HFpEF is challenging and supported by two score-based algorithms (H2FPEF and HFA-PEFF) which includes clinical, echocardiographic and blood laboratory values parameters.[6, 7]. Although HFpEF accounts for approximately 50% of all patients with HF, pathophysiological understanding and treatment options are limited [8–10]. There are a number of hypotheses on the pathogenesis of HFpEF, one of which being the assumption that diastolic dysfunction mainly, but not solely contributes to the development and course of HFpEF [11, 12]. Besides the functional patho-mechanisms, structural changes have been shown in patients with HFpEF: hypertrophic cardiomyocytes appear to be more frequent in HFpEF than in HFrEF [13] and collagen fraction is significantly increased compared to control persons [14]. There have been investigations concerning risk factors for the development of HFpEF, stating that age seems to be the most influencing, together with obesity, metabolic syndrome, sedentary lifestyle and arterial hypertension (aHT) [11].

The diagnosis of arterial hypertension relies upon accurate, reliable, and standardized blood pressure measurements. Indirect, cuff-based blood pressure measurements are typically performed with automated devices based on oscillometric techniques. Office blood pressure is the gold standard for diagnosis arterial hypertension, however, current guidelines recommend to confirm the diagnosis with out-of-office measurements such as ambulatory or home blood pressure monitoring with repeated measurements [15, 16]. In this review we focused on hypertensive response to exercise (HRE), as measured by using both automated and auscultation-based devices during physical effort while performing ergometry. HRE is defined as an exaggerated blood pressure response to exercise (EBPR), and has been shown to be related with a higher risk of development of future aHT [17–19]. No universally accepted definition of HRE exists today, however, several previous studies have suggested a systolic blood pressure elevation during exercise testing ≥ 210 mm Hg for men and ≥ 190 mm Hg for women or diastolic blood pressure elevation > 110 mm Hg in both genders [20–24]. According to the current European guidelines for sports cardiology, an exaggerated systolic blood pressure (SBP) to > 200 mm Hg at a workload of 100 W during exercise testing should prompt a clinical evaluation and optimization of antihypertensive medical therapy; whereas in young Olympic athletes a peak SBP > 220 mm Hg in males and > 200 mm Hg in females are beyond the 95th percentile [25]. In a recent review, our group described how HRE is not a normal finding in athletes and, as such, it might represent a risk factor for the future development of cardiovascular complication [26]. The current European guidelines for sports cardiology, although recognizing the under investigated problem of HRE, did not propose any definition for HRE [16].

Estimations about the prevalence of HRE range between 3–4% in a systematic review [27], 18% in a cohort of normotensive, young and healthy men and women [28], and 40% in a study of middle-aged, healthy men [29]. As with prevalence, pathophysiological processes and clinical implications of HRE are poorly understood.

It is the aim of this review to evaluate the available evidence for the hypothesis of a continuum of HRE, aHT and HFpEF.

## Methods

The systematic literature search was carried out using the PRISMA 2020 guidelines [30]. As for literature extraction, the PubMed database was searched for papers with the use of the following terms: “heart failure”, “diastolic heart failure”, “heart failure with preserved ejection fraction”, “hypertensive response to exercise”, “exercise hypertension”, “exaggerated blood pressure response”, “hypertension”, “arterial hypertension”, “systemic hypertension”. Only articles in English and studies/reviews who were done in humans were included.

Duplicates were systematically studied and removed from the analysis. In order to find the relevant articles, title and abstract were screened and read respectively.

**Fig. 1:**
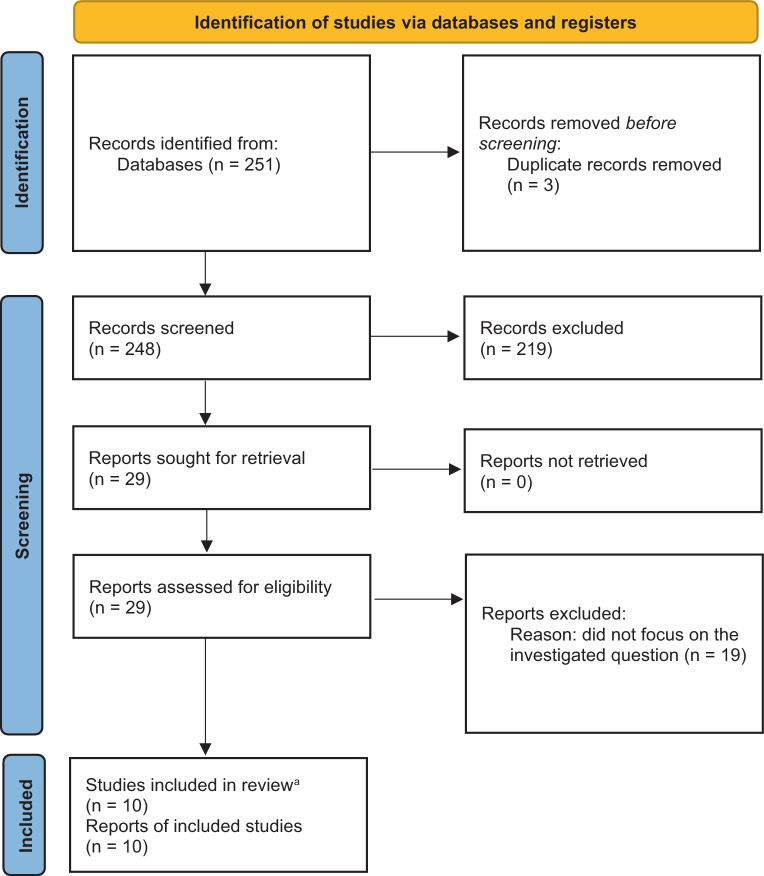
Flow of information, adapted from PRISMA guidelines, illustrated for HRE and aHT. After identifying 251 records, duplicates were removed, the remaining records were systematically screened, and 29 articles were elected for full-text analysis. Out of those, 10 were included in the final analysis. ^a^ One study included was a review and meta-analyses of literature and was therefore not included in the Table 1. *From:* [61]

**Fig. 2:**
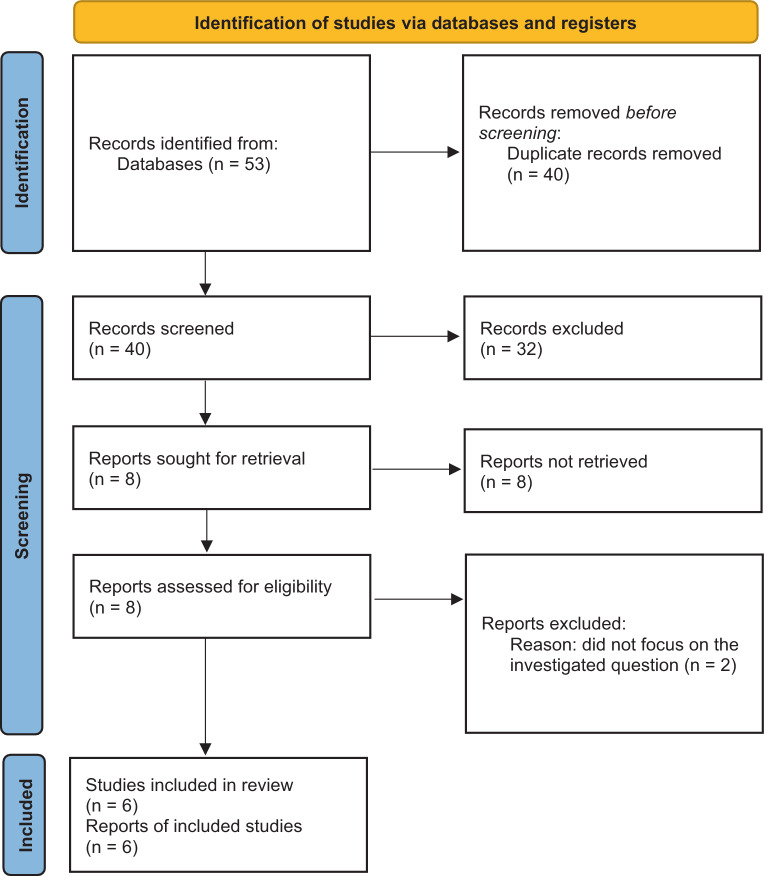
Flow of information, adapted from PRISMA guidelines, illustrated for HRE and HFpEF. 53 records were identified to fit into our analysis, whereof 40 remained after screening for duplicates. Another 32 records were excluded and at the end, 6 remained to be included in our final analysis. *From:* [61]

The literature search was implemented in three steps to connect the medical conditions with each other and find evidence for the continuum of disease: HRE and aHT, aHT and HFpEF, as well as HRE and HFpEF. As for the connection between aHT and HFpEF, we had no claim to completeness in this review as the association is well established [31, 32]. Also, a thorough and complete analysis on the pathogenesis of HFpEF with respect to aHT would have gone beyond the scope of this review. Hence, only major articles on this topic were included to demonstrate the association of aHT and HFpEF. Regarding HRE and aHT, the literature search yielded 251 papers. Out of those papers, 3 were excluded due to being duplicates and another 219 after checking the title and reading the abstract. Finally, 29 articles were classified as being relevant to the topic and 10 articles were found to be adequate to address our research question as whether HRE is a possible precursor of aHT (Fig. 1, Table 1). The literature search on a possible association of HRE and HFpEF yielded a total of 53 papers, out of which 13 were excluded being duplicates. Another 32 articles were excluded after screening the title and reading the abstract. Out of the 8 articles left for full-text analysis, 6 were found to be suitable to our research question (Fig. 2, Table 2). All relevant articles were read and included into this narrative review.

**Table 1:** Overview of clinical studies included in this review describing an association between Hypertensive Response to Exercise and aHT

Study	Year	Sample size	Subjects’ characteristics	Outcome parameters	Follow-up period	Outcomes
*Manolio TA et al.* [22]	1994	3741	Normotensive, young adults	aHT in future	5 years	Subjects (mainly black women) with HRE had a 1.7-fold increased risk for the development of future aHT
*Miyai N et al.* [35]	2000	239	High-normal BP, middle-aged	aHT in future	4.7 years	HRE was associated with an increased risk for future aHT (RR = 2.31, 95% CI = 1.45 to 6.25)
*Sharabi et al.* [39]	2001	190	Healthy male employees with a mean age of 42.6 years, without aHT, without use of antihypertensive drugs	aHT in future	5.7 years	Employees showing an HRE during exercise testing (defined as increase > 200 mm Hg in SBP or > 100 mm Hg in DBP) had a 5.64-fold increased risk to develop aHT (95% CI 1.40–22.30, P < 0.01)
*Farah et al.* [37]	2008	30	Healthy individuals aged from 20 to 64 with no cardiovascular disease, especially no resting aHT	aHT in future	2 years	84% of the individuals with HRE (SBP > 210 mm Hg) during stress exercise testing developed aHT
*Odahara et al.* [38]	2010	1008 (815 completed follow-up)	Individuals with no cardiovascular disease (no evidence of coronary heart disease, aHT)	aHT in future	7 years	Individuals with HRE had a HR of 2.264 (95% CI, 1.37–3.73) to develop aHT in the future, compared to normotensive individuals
*Lima et al. *[41]	2013	219	Cardiovascular healthy, without medications altering blood pressure values	aHT in future	3.5 years	Risk factors for developing aHT included older age, higher BMI. HRE was found **not** to be an independent risk factor
*Berger A et al. *[40]	2015	7082	Normotensive, middle-aged	aHT in future	5 years	BP response during exercise predicts future aHT
*Jae SY et al.* [34]	2015	3742	Normotensive, cardiovascular healthy	aHT in future	5 years	Peak SBP > 181 mm Hg (AUC = 0.644) and relative SBP > 52 mm Hg (AUC = 0.549) during exercise testing proved to be the most accurate cut-off values to predict future aHT
*Yzaguirre et al.* [36]	2017	107	Cardiovascular healthy, aged under 50 years	aHT in future	20 years	SBP > 180 mm Hg and/or DBP > 95 mm Hg proved to be best predictors for new onset aHT in the future

*Abbreviations: HRE* Hypertensive Response to Exercise, *aHT* Arterial Hypertension, *SBP* Systolic Blood Pressure, *DBP* Diastolic Blood Pressure

**Table 2:** Overview of clinical studies included in this review describing an association between HRE and HFpEF

Study	Year	Sample size	Subjects’ characteristics	Outcome parameters	Follow-up period	Outcomes
*Kato et al.* [49]	2008	40	Elderly patients with increased resting systolic aHT, LVH, LV-EF > 0.5, E/A < 1.0	BP-course, Plasma-Angiotensin and BNP-levels	4 weeks	SBP-increases were significantly higher in patients with DHF than in patients with aHT
*Takamura et al. *[50]	2008	129	Patients with LV-EF > 0.5, no evidence of myocardial ischemia or valvular heart disease	Electrocardiography, Course of HR, SBP, DPB during treadmill exercise testing	–	Impairment of left ventricular function was more present in patients with HRE than in control group
*Chung et al.* [53]	2017	797	Middle-aged patients without cardiomyopathy, valvular heart disease, pulmonary artery disease or LV outflow tract obstruction, but with frequent history of hypertension (75%)	Epidemiographic parameters like sex and age, as well as several indices using echo-Doppler evaluation, such as LVEF, LV mass index, LAVI, mitral inflow PW Doppler (amongst others)	–	HRE is more frequent in women and in both genders shows a consistent association with well-established risk factors of HFpEF
*Mottram et al.* [23]	2004	400	Middle-aged patients (men and women) with certain comorbidities, but without history of ischemic heart disease	LV diastolic function (pulsed wave Doppler imaging, LV color flow propagation, mitral annular diastolic velocity, etc.), structural measurements	–	No correlation between patients with HRE and LV hypertrophy. Patients with HRE and no coexisting hypertension showed high normal BP values
*Lee et al.* [52]	2014	118	118 hypertensive patients (61 men, 57 ± 11 years) were compared to 45 normotensive control subjects (16 men, 54 ± 8 years)	Clinical characteristics, CPET, echocardiographic and laboratory findings	–	LAVI was found to be an independent predictor of HRE in hypertensive patients but not in normotensive control subjects when controlled for age, sex, body mass index and peak oxygen consumption
*Vasan et al.* [54]	2001	9845	Participants in the Framingham Heart cohort. Aged 35–64 (*n* = 8244) and 65–94 (*n* = 1601). 24% had high-normal BP at baseline, the other participants had normal BP values	Progression to overt hypertension	4 years	Obesity and weight gain contributed to progression to hypertension; a 5% weight gain on follow-up was associated with 20–30% increased odds of hypertension

*Abbreviations: LVH* Left ventricular hypertrophy, *LV-EF* Left ventricular ejection fraction, *E/A* ratio used in assessing diastolic dysfunction, *BP* Blood pressure, *BNP* Brain natriuretic peptide, *SBP* systolic blood pressure, *DBP* Diastolic blood pressure, *DHF* Diastolic heart failure, *HR* Heart rate

## Results

### HRE and aHT

We report on a total of 10 studies including overall 16,165 subjects, investigating the association between HRE and future risk of developing aHT. These studies reported on a positive association between HRE and a higher risk of developing aHT as compared to individuals without HRE. The consistency of these findings were also confirmed in 17 out of 18 studies which were recently analysed in a systematic review studying the impact of HRE on the development of future aHT in normotensive individuals. According to this review, patients who manifested HRE during cardiopulmonary exercise testing (CPET) had a 1.4 to 4.2-fold higher risk of developing aHT [33].

Jae et al. went a step further and analysed the prognostic significance of blood pressure values analyzed as continuous variables with respect to the future development of aHT. They found that the most significant values for predicting future aHT were blood pressure values > 181 mm Hg or a relative SBP of 52 mm Hg during exercise testing (a relative SBP was defined as peak SBP minus resting SBP) [34]. These values are missed in the currently used definitions of HRE.

In the CARDIA study including 3741 young adults with no history of aHT, Manolio et al. also investigated the role of HRE and the development of aHT. These subjects underwent treadmill testing and performed a follow-up clinical visit after 5 years to evaluate for the development of aHT. HRE was defined as an increase of SBP > 210 mm Hg in men and > 190 mm Hg in women. 18% of all subjects met these criteria for HRE and, after a 5-years follow-up, presented with a 1.7-times higher likelihood to develop aHT as compared to subjects with normal SBP values during stress exercise. However, after stratification for race and gender, it turned out to be significant only in black women [22].

In 2000, Miyai et al. focused on 239 male subjects with high normal BP (SBP 130–139 mm Hg, diastolic blood pressure (DBP) 85–89 mm Hg). Subjects with any history of cardiovascular disease, stroke or diabetes or the usage of any drugs influencing BP were excluded. After a mean follow-up of 5.1 years, HRE turned out to be an independent risk factor for the development of aHT on multivariate analysis using the Cox proportional hazards survival model after adjusting for traditional risk factors (relative risk = 2.31, 95% confidence interval = 1.45 to 6.25) [35].

Yzaguirre et al. investigated the blood pressure thresholds for the development of late onset aHT in young patients (mean age 25.7 ± 11.1 years, 72% men) and investigated several cut-off values for HRE. The main differences between the six different definitions were whether the BP defining HRE was measured at maximum effort or at 100 watts. Statistically significant (odds ratio > 1 after adjustment for age) predictors were a maximal elevation of SBP values > 180 mm Hg at 100 Watts and DPB values > 95 mm Hg at peak exercise. Patients, whose values exceeded at least one of the two parameters were found to have a 70% increased risk of developing future aHT 20 years after the initial exercise testing [36].

In 2008, Farah et al. investigated 30 healthy individuals (no aHT, body mass index < 30 kg/m^2^ (BMI), normal lipid profile, no cardiovascular diseases and without known family history of hypertension), aged 20 to 64 years. A stress test using the Bruce protocol was performed and HRE was defined as a SBP > 200 mm Hg and an increase of DBP > 10 mm Hg during exercise testing. Additionally, another threshold, termed “hypertension in-exercise” (HIE), and defined as a SBP increase ≥ 200 mm Hg or DBP increase ≥ 100 mm Hg was investigated. Based on these parameters, the population was divided into 2 subgroups (17 normotensive vs. 13 hypertensive individuals). After two years of follow-up, individuals with HIE were more likely to develop aHT (defined as resting BP > 140/90 mm Hg) than their normotensive control group (77% vs. 6%, *p* < 0.001). Therefore, they concluded that abnormally high BP values during stress testing should be taken into account even in absence of aHT [37].

Odahara et al. published a prospective study in 2010 investigating the influence of potential risk factors for the development of aHT in 815 subjects with no cardiovascular disease and normal electrocardiogram results at baseline. HRE was defined as SBP > 250 mm Hg or DBP > 120 mm Hg. Over a mean follow-up of 7 years, 13.3% subjects developed aHT. An independent association between HRE and aHT was confirmed in multiple cox hazard analyses (hazard ratio: 2.26 (95% CI 1.34–3.73)) [38].

Sharabi et al. investigated 2783 healthy Israel Defense Force employees aged > 26 years in the setting of a periodic medical evaluation. Every employee aged > 39 years underwent an exercise stress testing with Bruce protocol. HRE was defined as an increase in SBP > 200 mm Hg or DBP > 100 mm Hg during exercise. After excluding women, subjects with known aHT and/or taking antihypertensive drugs, data from 190 male subjects (mean age 42.6 years) were analyzed. Out of the 190 subjects, 73 showed HRE. After a mean follow-up period of 5.7 years, patients with HRE had a 5.64 odds ratio (95% CI 1.40–22.30, *p* < 0.01) of developing aHT after adjustment for confounding factors [39].

Berger et al. investigated 7082 individuals (mean age 48 ± 9 years) subjects with > 4 consecutive exercise testing with BP measurement available and followed them up for 5 ± 3 years. Subjects with a previous diagnosis of aHT, on antihypertensive drugs, and history of cardiovascular disease were excluded. In a univariate Kaplan-Meier survival analysis, a cumulative probability (35%, *p* < 0.001) of future aHT for the highest quintile of SBP during exercise was found. Similar findings were described for DBP values. In a multivariate Cox proportional hazards regression modeling, subjects in the highest quartile of exercise SBP had a 2.58-fold (*p* < 0.001) risk of developing aHT. Each 5 mm Hg increase in exercise SBP and DBP were independently associated with, respectively, a 11% and 30% increased risk for new onset of aHT [40].

In contrast to the above mentioned studies, Lima et al. did not find any correlation between HRE and aHT and propose that HRE found in patients should rather be considered as an intermediate state between normotensive BP values and clinically established aHT [41].

To summarize, several studies convincingly show that HRE might be a possible intermediate condition in the development of aHT.

### aHT and HFpEF

The primary patho-mechanism beyond the evolution from hypertension to HFpEF is based on the LaPlace’s law. Indeed, aHT results in a greater left ventricular pressure that triggers wall thickening in order to reduce wall stress. Consequently, left ventricular hypertrophy (LVH) develops. As a consequence, LVH leads to further myocardial impairments due to, among others, myocardial ischemia caused by a shortage of myocardial blood supply, especially in the midmyocardial layer [42, 43]. This, in turn, results in a fibrotic remodelling and stiffening of the ventricle, promoting elevated left ventricular (LV) filling pressures and therefore ensuing LV diastolic dysfunction [5, 44]. In support to the positive relationship between aHT and HFpEF, Redfield et al. showed that aHT clearly induced vascular remodelling and increased arterial stiffness in a subpopulation of patients particularly at risk for HFpEF (e.g. elderly and women) [45, 46]. Other factors, such as inflammation, might lead to coronary microvascular endothelial inflammation which might trigger LV hypertrophy [47, 48]. In summary, aHT plays an important role in the pathogenesis of HFpEF.

### HRE and HFpEF

We report on a total of 6 studies including overall 1366 subjects, investigating the association between HRE and HFpEF. In the early 2000s, the definition of HFpEF as recently proposed in the current guidelines had not been developed, yet, and the condition we refer to HFpEF today, was referred to as diastolic heart failure. This needs to be taken into account when older studies investigating the association between HRE and HFpEF are analyzed.

As such, Kato et al. investigated 20 patients with diastolic heart failure and 20 age-matched (70 ± 4 years) hypertensive patients with LVH with no differences in BP values at rest and left ventricular (LV) mass. Despite similar relevant baseline characteristic, patients with diastolic heart failure had significantly higher SBP during exercise testing as compared to the control group. Mechanistically, it was suggested that ventricular and arterial stiffness, altered atrial constriction, different preload reserves and higher LV afterload in patients with HRE resulted in further deterioration of myocardial relaxation and LV filling [49].

Takamura et al. investigated 129 patients with coronary artery disease undergoing exercise test and divided them into three age-matched groups: HRE (defined as HRE with normal BP at rest), HRE + aHT, and a control group without normotensive values both at rest and during exercise. In this study, HRE was defined as SBP/DBP ≥ 210/105 mm Hg in males and ≥ 190/105 mm Hg in females, as measured after 6 min of exercise testing using the modified Bruce protocol. On echocardiography, subjects included in the groups HRE and HRE + aHT presented with significantly lower e’ values (e.g. early diastolic mitral annular velocity) and a significantly higher E/e’ values (which is a surrogate parameter reflecting LV filling pressure) [50].

In a previous, meta-analysis including 12 studies and 46.314 individuals which were followed-up for 15.2 ± 4.0 years, Schultz and al. found a 36% higher rate of adverse cardiovascular events (defined as nonfatal and fatal myocardial infarction, nonfatal or fatal cerebrovascular events or the development of coronary artery disease) and mortality in patients with HRE in comparison to subjects without HRE [51].

Lee et al. investigated the relevance of left atrial volume index (LAVI) in 118 hypertensive and 45 normotensive individuals and found it to be an independent predictor of HRE in hypertensive individuals but not in the normotensive control group after controlling for body mass index, age, gender and peak oxygen consumption [52].

Chung et al. investigated data from 797 patients (38% women, mean age 64 ± 10 years, 75% with a history of hypertension) and showed that the prevalence of HRE was higher in women than in men. Furthermore, patients with HRE presented with pathologic variations in LV mass index, LAVI, E/e’, end diastolic elastance and relative wall thickness, which are echocardiographic parameters of diastolic dysfunction and ventricular remodelling and known risk factors for the development of HFpEF [53].

Mottram et al. investigated 400 patients who initially presented with chest pain and underwent a cardiological work-up including exercise stress test, electro- or echocardiography and compared them to 17 gender- and age-matched controls [23]. Among the patients, 41 had HRE (defined as > 210/105 mm Hg in men; > 190/105 mm Hg in women), 22 presented with resting hypertension (HRE + aHT), and 19 were normotensive [23]. In this study, a high-normal resting BP (mean 136/86 mm Hg) was detected in the HRE-group. Such high-normal resting BP values represent a risk factors for the development of future aHT and, consequently, of HFpEF [54]. Controversially, no association was found between HRE and LV hypertrophy after correcting for confounding factors. The authors assumed that previous studies showing such correlations used less sensitive measurement methods to assess diastolic and systolic function [23].

## Discussion

This review article aimed to investigate possible associations i.e. a continuum of disease of HRE, aHT and HFpEF. Indeed, if such a continuum exist, HRE would be the earliest detectable pathology in the long term development of HFpEF and therefore would have huge clinical implications.

### Necessity for a definition of HRE

Until today, no universally accepted definition of HRE has been determined. The definitions of HRE in the presented studies vary considerably (Table 3) and do not propose a clear threshold between health and disease, or “not-at-risk subjects” and “high-risk individuals”. Jae et al. and Yzaguirre et al. both found in their studies that SBP-values during exercise testing below the currently proposed thresholds (> 181 mm Hg SBP and > 180 mm Hg respectively) already proved to be significant as a predictor for the development of future aHT [34, 36]. These data imply the urgent need for a universally and scientifically justified definition of HRE based on its possible pathological consequences.

**Table 3:** Overview of the inconsistency concerning the definition of HRE

Reference	Cut-offs used or proposed (each during exercise testing)
Allison TG et al. [15], Lauer MS et al. [16], Manolio TA et al. [17]	Absolute SBP elevation ≥ 210 mm Hg in men Absolute SBP elevation ≥ 190 mm Hg in women
Mottram PM et al. [18]	Absolute BP elevation > 210/105 mm Hg in men Absolute BP elevation > 190/105 mm Hg in women
Schultz MG et al. [19]	Absolute SBP elevation ≥ 210 mm Hg in men Absolute SBP elevation ≥ 190 mm Hg in women *or* Absolute DBP elevation > 110 mm Hg in both genders
Jae SY et al. [28]	Absolute SBP elevation > 181 mm Hg in both genders *or* Relative SBP elevation > 52 mm Hg in both genders
Sharabi Y et al. [35]	Absolute SBP elevation > 200 mm Hg in *men or* Absolute DBP elevation > 100 mm Hg in men

### Individuals with HRE are at risk

Several studies demonstrated a positive association between HRE and the new onset of aHT. Keller et al. performed a systematic review and concluded that 17 out of 18 included studies pointed towards HRE being a risk factor for the development of future aHT (relative risk: 1.4–4.2) [33]. These findings have been confirmed in smaller studies [35, 37–39]. The heterogeneity of statistical methods as well as the use of different cut-off values for the definition of HRE, the different population characteristics and study design renders it difficult to draw absolute conclusions. However, all studies confirmed the role of HRE as risk factor for the future development of aHT.

### Systolic or diastolic parameters?

During exercise, SBP physiologically increases, whereas DBP drops or does not increase significantly as sign of peripheral muscular vasodilation under effort [27]. Having that in mind, one could tend to search and define pathologies according to abnormalities in the increase and course of SBP during exercise. However, Yzaguirre et al. found that besides the expected importance of SBP abnormalities, a maximal elevation of DBP > 95 mm Hg at peak exercise was significantly associated with the development of future aHT [36]. Moreover, Berger et al. reported on the importance of DBP response, stating that individuals with exercise DBP in the top quartile turned out to be at highest risk to develop aHT in the future, and DBP was even more predictive than SBP [40]. These conclusions are important and, as such, DBP values are also included in most all definitions of HRE. However, according to the current European guidelines for the management of aHT, DBP cannot be reliably measured non-invasively during exercise [55]. Of note, DBP was assessed non-invasively in the two above-cited studies. Hence, data describing an association between alterations in DBP response and aHT in the future should be interpreted very carefully. Of course, an invasive BP monitoring during exercise is difficult and not feasible in clinical practice—however, the evidence of a link between HRE, aHT and HFpEF, highlight the importance of DBP and should prompt further research to measure it more rigorously.

### aHT and HFpEF

The pathophysiology of HFpEF is still not fully understood due to its very complex nature. Nevertheless, aHT has long since been identified as one of the most important risk factors for the development of HFpEF. There are various pathophysiological factors linking aHT and HFpEF, one of which being the long-known assumption that vascular and ventricular stiffening (as a consequence of aHT) greatly influence the onset of HFpEF [45, 46]. However, as highlighted in more recent studies, the development of HFpEF is not only the result of mechanical changes due to long-standing aHT [47]. Indeed, there are multiple, previously unknown, non-mechanical correlations, where further research has to be conducted in the future. This could open new research opportunities for new therapeutic targets in order to improve our repertoire of therapies in patients with HFpEF.

### HRE and HFpEF

Up to now, only few studies investigating the influence of HRE on HFpEF have been published. Currently, there is no study showing a direct, causative connection between patients presenting HRE and the future development of HFpEF. However, several studies suggest that such a connection exists: Kato et al. showed that an HRE occurred more often in patients suffering from HFpEF than in patients having similar blood pressure values at rest and comparable LV mass [49]. However, these findings do not directly provide evidence about our hypothesis of the role of HRE as an early detectable abnormality in patients with HFpEF, since the patients presenting with HRE included in the study were already diagnosed with HFpEF. Nevertheless, these findings confirm that the two conditions are, in a way, connected. Moreover, Takamura et al. concluded that in patients with HRE E/e’ values were significantly higher as surrogate parameter of elevated LV filling pressure and impaired LV diastolic function independently from the presence of aHT [50]. LV diastolic dysfunction is considered as precursor of HF [1]. Hence, these results could possibly indicate a pathophysiological link (e.g. the presence of LV diastolic dysfunction) between patients with HRE and patients suffering from HFpEF. Additionally. Schultz et al. published a systematic review stating that patients with HRE have a 36%-higher risk of developing adverse cardiovascular events, one of which being CAD [51]. CAD is common in patients with HF and has been identified as risk factor [8, 32, 56–58], suggesting another link between HRE and HFpEF.

### Limitations

This review has several limitations. Firstly, limited long-term follow-up and only small study populations on the topic limited an exact investigation of the correlation between HRE, aHT and HFpEF. Up to now, only few studies dealing with long-term cardiovascular outcomes of patients with HRE have been conducted [51]. Indeed, despite 219 papers were screened, only 10 could be evaluated for the current study question and, therefore, some aspects of hypertensive response might not have been fully captured. We could only include original studies performed between 1994 and 2017, so newer studies are needed to further investigate this study question in the light of new therapies. Secondly, there is a lack of definitions concerning HRE itself, which makes it difficult to compare different studies. Namely, thresholds differ over the years and are, up to the present day, not universally defined in official guidelines [20–23]. Thirdly, aHT is only one of many reasons why HFpEF develops [46]. This heterogeneity of pathological mechanisms leading to HFpEF makes it difficult to investigate the correlation between one element (in our case aHT) and HFpEF as an isolated consequence with respect to the course of the disease. Additionally, aHT itself underlies multiple risk factors and pathophysiological mechanisms—HRE is one of those [33, 34, 36]. Hence, the assumption of a direct correlation between HRE, aHT and HFpEF is utterly difficult to investigate, since all three conditions themselves are being influenced by a great variety of factors.

## Conclusion

**Fig. 3:**
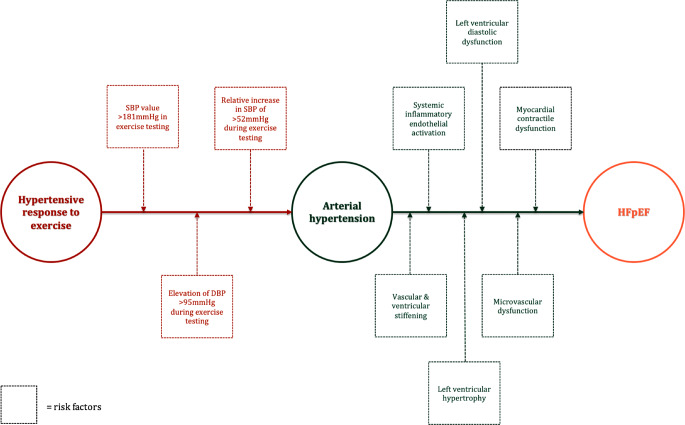
Timeline and selection of risk factors in a possible course of disease. Patients, in which HRE has been detected and whose values during stress testing exceeded absolute SBP values > 181 mm Hg, absolute DBP values > 95 mm Hg or relative elevations in SBP > 52 mm Hg seem to be most at risk to develop aHT. This in turn can lead to the development of HFpEF

In conclusion, there is coherent data on the association between HRE and aHT, as well as between aHT and HFpEF—however, long-term data investigating the continuum of disease is scarce (yet promising) and further research is needed. Our comprehensive literature search revealed suggestive data concerning a connection between all three conditions: patients with HRE are at risk of developing aHT [33–35, 37–39], aHT itself is known to be one of the main risk factors for the development of HFpEF [46, 59, 60], and HRE being linked to diseases at risk of ending in HFpEF, such as LV diastolic dysfunction and CAD [50, 51, 56–58]. aHT represents the principal risk factor for the development of HFpEF and, as such, has been intensely studied. Early findings, as well as newer insights, consequently underline the fact that aHT can progress to HFpEF over time. Figure 3 shows a summary of patho-mechanisms and a possible course of disease. Hence, the key question seems to be to which extent HRE in otherwise young and healthy individuals poses a risk to progress to aHT in the future. In this review, we have reported on several studies which support such an association. However, uncertainties still remain: How should HRE be defined? Should we focus more intensely on measuring DBP during exercise, rather than on SBP? Gender and ethnic aspects should be further elucidated in future studies. Long-term prospective studies investigating cardiovascular outcomes in patients with HRE are needed to address these questions and corroborate our hypothesis of a continuum of disease.
